# An Introduction to Epidemiologic and Statistical Methods Useful in Environmental Epidemiology

**DOI:** 10.2188/jea.JE20100010

**Published:** 2010-05-05

**Authors:** Hiroshi Nitta, Shin Yamazaki, Takashi Omori, Tosiya Sato

**Affiliations:** 1 Environmental Health Science Division, National Institute for Environmental Studies, Tsukuba, Ibaraki, Japan; 2 Department of Epidemiology and Healthcare Research, Kyoto University School of Public Health, Kyoto, Japan; 3 Department of Biostatistics, Kyoto University School of Public Health, Kyoto, Japan

**Keywords:** design and analysis, environmental epidemiology, methodology

## Abstract

Many developments in the design and analysis of environmental epidemiology have been made in air pollution studies. In the analysis of the short-term effects of particulate matter on daily mortality, Poisson regression models with flexible smoothing methods have been developed for the analysis of time-series data. Another option for such studies is the use of case–crossover designs, and there have been extensive discussions on the selection of control periods. In the Study on Respiratory Disease and Automobile Exhaust project conducted by the Japanese Ministry of the Environment, we adopted a new 2-stage case–control design that is efficient when both exposure and disease are rare. Based on our experience in conducting air pollution epidemiologic studies, we review 2-stage case–control designs, case–crossover designs, generalized linear models, generalized additive models, and generalized estimating equations, all of which are useful approaches in environmental epidemiology.

## INTRODUCTION

What is environmental epidemiology? How can environmental epidemiology be differentiated from other subspecialties of epidemiology? According to the current edition of A Dictionary of Epidemiology, environmental epidemiology^1^ is defined as the “branch or subspecialty of epidemiology that uses epidemiological principles, reasoning, and methods to study the health effects on populations of exposure to physical, chemical, and biological agents external to the human body and of immediate and remote social, economic, and cultural factors (eg, urbanization, agricultural development, energy production/combustion) related to these physical, chemical, and biological agents.” However, this definition is not specific to environmental epidemiology.

The definition of environmental epidemiology in a textbook edited by Steenland and Savitz^2^ invokes a key concept to contrast environmental epidemiology from other subspecialties of epidemiology. They suggest that “environmental epidemiology may be defined as the epidemiologic study of the health consequences of exposures that are involuntary and that occur in the general environment.” The key word here is “involuntary.” If we adopt this definition, as shown in their textbook, passive smoking is within the scope of environmental epidemiology, whereas active smoking is not. This core characteristic of environmental epidemiology, ie, involuntary exposure in the general environment, creates difficulties in exposure assessment in the field of environmental epidemiology. There is great diversity in the manner of exposures in human populations and considerable space–time variability, which is influenced by several external conditions. Various study designs and methods of data analysis have been developed in the process of overcoming these difficulties.

In this review, we explain several recent methodological developments. Some of these developments arose in the field of environmental epidemiology and later crossed disciplinary boundaries. The generalized estimating equations method, for example, was developed to analyze repeated measurements of health effects associated with air pollution, but is now used widely in the social sciences, as well as in the health sciences.

We have recently utilized these designs and statistical methods for studies on health effects and air pollution. A 2-stage case–control design was used for the SORA (Study on Respiratory Disease and Automobile Exhaust) project, which is a group of epidemiologic studies of infants, schoolchildren, and adults. Although many epidemiologic studies have shown associations between traffic density and asthma prevalence or morbidity, few have examined the relationship between asthma incidence and traffic-related exposures. Well-designed studies are needed to assess the question of whether exposure to traffic-related air pollutants is a risk factor for the onset of asthma or other respiratory illnesses in children and adults. For this purpose the Japanese Ministry of the Environment developed SORA.

Both the case–crossover design and generalized additive models are used extensively as powerful tools for analyzing the relationship between short-term exposure to air pollutants and health effects. Generalized additive models are the standard technique for time-series analyses that assess the health effects of environmental variables. Generalized additive models do not assume linearity and allow for more flexible analyses using nonparametric smoothing. A case–crossover design is conducted to evaluate the transient effects of time-varying exposure on events. With this design, only cases are analyzed, and within-individual comparisons eliminate the effects of confounding, which do not change long-term. This method has become popular in analyzing the acute effects of air pollutants on health.

Based on our experience in conducting air pollution epidemiologic studies, we review 2 new study designs—2-stage case–control and case–crossover designs—and 3 new statistical methods—generalized linear models, generalized additive models, and generalized estimating equations—which have proven to be useful tools in environmental epidemiology.

## TWO-STAGE (2-PHASE) CASE–CONTROL DESIGNS

We were involved in the design, conduct, and analysis of the ongoing SORA project. In the design of the infant case–control study of asthma and traffic air pollution, we set parameter values to calculate the required number of cases: the proportions of infant living near roads were 3% and 4%, the odds ratios for detection were 1.5 and 1.7, the case:control ratio was 1:2, and the 1-sided alpha level was 5%. Table 1 shows the required number of cases.

**Table 1.** Required numbers of cases in a case–control study of childhood asthma and traffic air pollution

	Odds ratio = 1.5		Odds ratio = 1.7	
Proportion living near roads	3%	4%	3%	4%

Power				
90%	2320	1766	1297	989
80%	1684	1282	943	719
70%	1292	984	725	553
60%	1000	762	563	430
50%	765	583	432	330

A study with at least 80% power would have required more than 1000 cases and at least 3000 study participants. Why were such large numbers required? Because both the disease under study (childhood asthma) and the exposure (proportion of infants living near roads) were rare. In such a case, neither a cohort nor a case–control study is efficient.

A 2-stage case–control design has been proposed for the study of the relationship between a rare exposure and a rare disease.^3^ In the first stage of a 2-stage case–control design, we identify cases and controls and obtain their exposure information, as shown on the left side of Table 2. In the second stage, we obtain random samples from each of the 4 cells in the first stage, with sampling probability *s_ij_* (*i* is a case index: *i* = 1 if cases, *i* = 0 otherwise; *j* is an exposure index: *j* = 1 if exposed, *j* = 0 otherwise), as shown on the right side of Table 2. Because exposed cases are more informative than others, *s*_11_ is often set to 1. For other cells, a balanced design to choose nearly equal numbers as *n*_11_, may have good efficiency (ie, a smaller standard error for an odds ratio).^4^

**Table 2.** Data layout for a 2-stage case–control design

	First stage		Second stage	
		
	Exposed	Unexposed	Exposed	Unexposed
Cases	*N*_11_	*N*_10_	*n*_11_	*n*_10_
Controls	*N*_01_	*N*_00_	*n*_01_	*n*_00_

We then obtain more detailed information on exposure and important covariates for subsamples only in the second stage. In estimating covariate adjusted odds ratios, we need special analytic methods that take into consideration the sampling structure in the second stage. We assign a weight to each second-stage participant as an inverse probability of sampling, 1/*s_ij_*. For example, when *s*_10_ = 0.1, the number of unexposed cases in the first stage would be 10 times higher. Weighted analyses with an inverse probability of sampling weights give an unbiased estimation of odds ratios by means of stratified analyses or logistic regression models.^3^^,^^5^^,^^6^ Although we know the true sampling probabilities, estimated weights substantially increase efficiency when we use the first-stage information in the estimation of weights.^7^

The 2-stage case–control design is useful when both disease and exposure information is relatively easily obtained. In the SORA project case–control study, we determined asthma incidence by using the standard questionnaire for preschool children, and estimated elemental carbon concentration outside a participant’s house by using their address and mapping information. Detailed information, such as personal exposure estimation, life history, and blood sampling data were obtained only from second-stage subsamples.

## CASE–CROSSOVER DESIGNS

In the first half of the 20th century, several episodes of extreme air pollution focused attention on the potential adverse health effects of air pollution. These included an episode in the Meuse Valley, Belgium, in December 1930,^8^ one in London, England in 1952,^9^ and another in Yokkaichi, Japan in the 1960s.^10^ Episodes such as the one in London in 1952 resulted in a clear increase in the number of deaths during the period concurrent with the pollution episodes.^9^ The evidence that acute air pollution episodes cause short-term increases in mortality is persuasive. Thus, we have an anchor of certainty that air pollution causes adverse health effects in the high-dose region of the dose–response curve.^11^^,^^12^

A much greater challenge is to determine whether an analogous phenomenon occurs within the much lower range of air pollution routinely experienced in urban areas of modern societies.^12^ Such a research question arises directly out of these historical acute episodes. Moreover, repeated, persistent, small adverse effects in large populations have the potential for sizable public health consequence. Some studies have addressed the possible effects of long-term exposure to air pollution on the development of chronic diseases such as lung cancer and cardiovascular disease, but the current and most intensively pursued research avenue is the short-term effects of pollutant levels on mortality and morbidity, and the etiologic process operating on a time scale of days rather than years.^12^ There remains disagreement as to the level of pollution that would significantly affect human health.

The case–crossover design is an attractive approach to examine the impact of time-varying exposures that may be triggers of adverse health events.^13^ It has been used, for example, to investigate triggers of myocardial infarction^14^ and road-traffic accidents.^15^ The case–crossover design requires exposure data for cases only. It can be regarded as a variant of the case–control study in which each case serves as his or her own control. In recent years, this design has been applied to the analysis of the acute effects of environmental exposures, especially air pollution, because it has the advantage of controlling for seasonal variation, time trends, and potential confounding caused by fixed or slowly time-varying confounders such as sex, race, diet, and age. For example, to examine the association between short-term outdoor air pollution and adverse health effects, a case–crossover study was conducted in a primary care clinic in greater Tokyo, Japan.^16^ In this study of children, the odds ratio in warmer months per 10-ppb increment in the 24-hour mean concentration of ozone was 1.16 (95% confidential interval [CI]: 1.00–1.33) after adjustment for temperature, and 1.29 (1.08–1.55) after adjustment for particulate matter less than 2.5 microns in aerodynamic diameter (PM_2.5_), NO_2_, and temperature.

**Figure 1.**
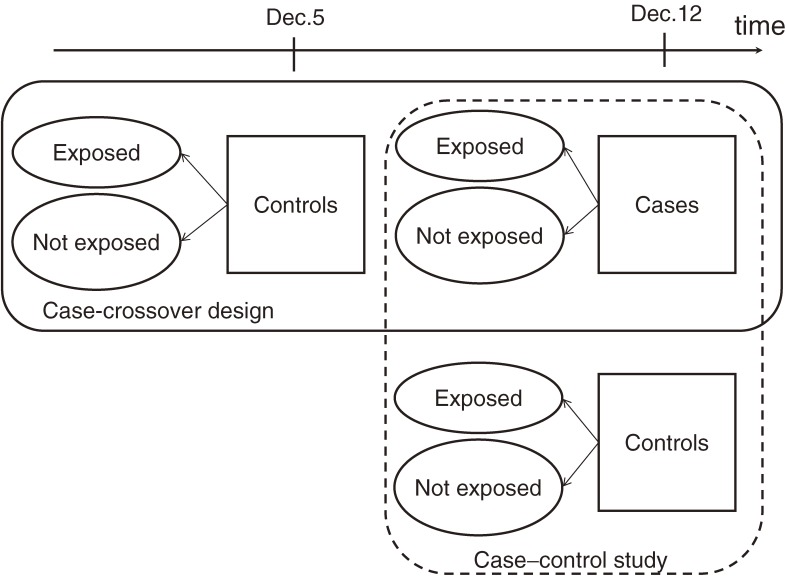
Designs of a case–control study and a case–crossover study

In the case–crossover design, a case–control study is conducted so that each person’s case period (when the event occurred) is matched with his/her previous time period (the control period) when he or she did not have the event (Figure 1). The subject's characteristics and exposures during the case period are compared with those during the control period. Each risk set consists of 1 individual as he/she crosses over between different exposure levels in the interval between the 2 time periods. These matched pairs may be analyzed using conditional logistic regression. Multiple control periods may be used.

**Figure 2.**
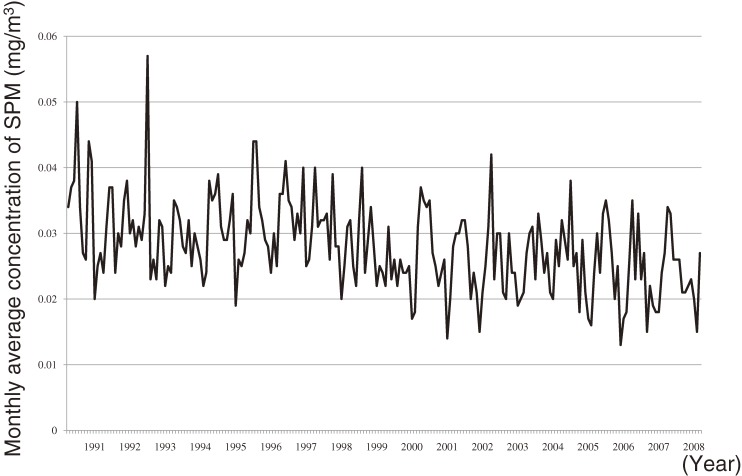
A graph of the concentration of suspended particulate matter (SPM) showing a long-term trend and seasonality. These monthly measurements of the mean concentration of SPM at Mibu, Kyoto, Japan were obtained from the National Institute for Environmental Studies, Tsukuba, Japan.

**Figure 3.**
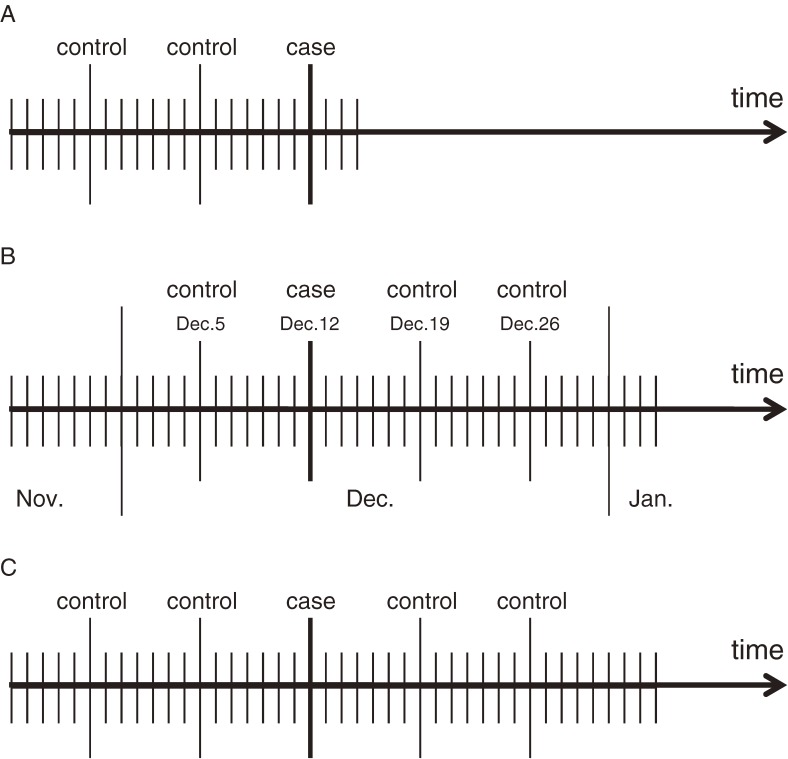
Example of control selection strategies. (A) Retrospective fixed-interval control selection; (B) Time-stratified case crossover design; (C) Ambidirectional fixed-interval control selection.^17^

Although this approach automatically controls for time-invariant confounders by design, it may allow selection bias and confounding by time-varying factors. For example, when concentrations of pollutants decrease with time, and if control periods were selected only retrospectively, the odds ratios would be biased toward null (Figure 2 and 3A). The time-stratified case–crossover design is one technique for minimizing bias in such a case (Figure 3B).^16^^,^^17^ Time stratification refers to the method by which the control periods are chosen. Specifically, time is stratified into months to select the days of control periods that fall on the same day of the week within the same month as the date that a health event occurred (the day of the case period). For example, if death occurred on Sunday, December 12, the 3 control days would be December 5, 19, and 26. The control periods can also be matched by the clock hour of case periods. Therefore, this approach also controls for long-term trends, seasonality, day-of-the-week effects, and circadian rhythm. The alternative strategy selects controls symmetrically among lags and leads of, say, 7 and 14 days (Figure 3C), and is referred to as a symmetric ambidirectional case–crossover design.^18^

When applied to the association of air pollution with the risk of death, the case–crossover design has several advantages. First, it clarifies a key feature of the study of acute response to air pollution because, in this type of analysis, each subject serves as his/her own control. Thus, the use of nearby day as the control period means that all covariates that change slowly over time, such as smoking history, age, body mass index, usual diet, diabetes status, etc, are controlled for by self-matching. Second, because the case–crossover design focuses on individual events, rather than daily counts, it permits the straightforward examination of effect modification. Third, the case–crossover design allows investigators to control for seasonal variations in mortality or morbidity risk. The other technique to analyze the association of short term changes in air quality with short term changes in the risk of death or hospital admission has been to collapse the data to daily counts, and use Poisson regression of the daily data. Because these regressions make comparisons across the full range of data, including multiple years, it is necessary to control for season and long-term time trends.

While it is straightforward to sample control days in a manner that removes seasonal confounding, selection bias is possible in these analyses. For example, the result of an ambidirectional case–crossover study to examine the transient effects of air pollution on the risk for onset of myocardial infarction would result in bias, because there would be a carry-over effect of treatment.

By design, the case–crossover design controls for seasonal variation, time trends, day-of-the-week effects on air pollution exposure, and slowly time-varying confounders because the case and control periods in each risk set are separated by a relatively short time interval.

## GENERALIZED LINEAR MODELS

In epidemiologic studies, regression modeling is a common approach for adjusting confounding factors. Because the ordinary linear regression model is the basis of regression modeling, we first consider a linear model. As an example, consider the hypothesis that people who live in an area with a higher average PM_2.5_ concentration will have a lower percent predicted forced expiratory volume in 1 second (PPFEV1). Since smoking is a well-known confounding factor in such a study, we adjust for pack-years, where 1 pack-year is defined as 20 manufactured cigarettes (1 pack) smoked per day for 1 year, using the following linear model:

$$\mathrm{PPFEV1}=\beta_{0}+\beta_{1}\mathrm{area}+\beta_{2}\mathrm{pack-years}+\varepsilon ,$$

where β_0_, β_1_, and β_2_ are intercept and regression coefficients, “area” is an indicator variable (area = 1 if subject lives in an area with a higher PM_2.5_ level; otherwise, area = 0) and ε is an error term. We assume that ε follows an independent and identical distribution with mean = 0 and variance = σ^2^, and regression coefficients are estimated by fitting a model to data under the assumption of error distribution. The right hand side of the above model has 2 essential parts: the structural model—β_0_ + β_1_ area + β_2_ pack-years—and the error term, ε.

Under the assumption for the error term, the expected PPFEV1, μ, is written as

$$\mu =\beta_{0}+\beta_{1}\text{area}+\beta_{2}\mathrm{pack-years}.$$

For the same number of pack-years, mean PPFEV1 for area = 1 would be β_0_ + β_1_ + β_2_ pack-years and mean PPFEV1 for area = 0 would be β_0_ + β_2_ pack-years. In other words, β_1_ is interpreted as the difference between mean PPFEV1 according to area, adjusted for pack-years. Note that this approach assumes that the response variable, PPFEV1, is continuous, and that it decreases (or increases) linearly with pack-years.

In general, the ordinary linear model with covariates X_1_,…, X*_p_* can be written as follows:

$$\mu =\beta_{0}+\beta_{1}X_{1}+\beta_{2}X_{2}+\cdots +\beta_{p}X_{p},$$

where effects of covariate Xs contribute additively.

Let us consider a study of the short-term effects of particulate matter on daily mortality. Typically, daily mortality counts from vital statistics in a specific area, and values for air pollutants and meteorological data measured at a representative monitoring station in that area, are arranged as shown in Table 3. To investigate the short-term effect of particulate matter on daily mortality adjusted for both temperature and humidity, a regression model is often used.^19^

**Table 3.** A hypothetical data layout for a study of the short-term effects of particulate matter on daily mortality

Day	No. of deaths	PM_2.5_ (µg/m^3^)	Temperature (°C)	…
1	18	23.6	9.3	
2	16	23.8	13.2	
3	16	22.2	13.7	
4	20	18.8	11.3	
5	11	23.1	13.8	
6	21	28.6	15.4	
…	…	…	…	…

Abbreviation: PM_2.5_, particulate matter less than 2.5 microns in aerodynamic diameter.

One modeling issue is that the response variable, daily mortality, is count finite and not continuous. When a response variable is a count, such as daily mortality, we may assume that it has a Poisson distribution. The other issue is that ratio measures, such as risk ratio, rate ratio, and odds ratio, rather than difference measures, are often used in epidemiologic applications. Generalized linear models (GLIM) extend the ordinal linear model to noncontinuous response variables.^20^ In GLIM, we assume a linear model for a functional transformation, g, of expectation, μ, as

$$g(\mu )=\beta_{0}+\beta_{1}X_{1}+\beta_{2}X_{2}+\cdots +\beta_{p}X_{p}.$$

In this model, the function, g, is referred to as a link function, and this function determines the relationship between the expected value, μ, of the response variable and linear combinations of X terms. When a response variable follows a Poisson distribution with mean = μ = *Mr*, where *M* is a person-time and *r* is an event rate and the rate ratio is a preferred measure of association, the natural log function is used as g. This model is known as the Poisson regression model. The rate ratio of the *j*th variable is expressed as exp(β*_j_*), while each subject has a term log *M* instead of β_0_ (to estimate rate ratios in statistical software, an option called offset is required).

When a response variable follows a binomial distribution with mean = μ = *p*, where *p* is the probability of an event, and log(*p*/(1 − *p*)) is selected as a link function, the model expresses the logistic model. In the logistic model, the odds ratio for the *j*th variable is expressed as exp(β*_j_*). Iterative calculations may be required to obtain estimates of coefficients in GLIM.

## GENERALIZED ADDITIVE MODELS

Another issue in regression modeling is the flexible representation of the relationship between a response variable and covariates, since we do not know their correct functional forms. The ordinary linear model assumes a simple linear relationship. We are not certain of the functional forms of relationships in epidemiologic research, and would like to avoid the substantial effect of functional form assumptions. The linearity assumption is simple, but sometimes too strong. One solution is the use of flexible nonparametric functions. An additive model is a typical example of such a model.

Ordinary linear regression assumes a linear relationship. The simplest case is that of a single covariate:

$$\mu =\beta_{0}+\beta_{1}X.$$

This expression shows that the relationship between μ and X is linear. Let us assume a data-dependent relationship and fit the more flexible function to the data. Smoothing techniques such as spline functions or kernel smoothers can help in achieving this requirement. We describe the model as follows:

μ=s(X).

Note that we need to specify smoothing (tuning) parameters that determine the smoothness of function *s*.

In the same manner, an additive model is constructed using the additive components of the flexible functions *s_j_*(X*_j_*):

$$\mu =\beta_{0}+s_{1}(X_{1})+s_{2}(X_{2})+\cdots +s_{p}(X_{p}).$$

To estimate the parameters included in each *s_j_*, iterative calculations, such as the backfitting algorithm, are needed.

Generalized additive models (GAM)^21^ allow a response variable to be a count, binary, or continuous variable, by assuming the appropriate distribution of a response variable, as in GLIM, and the additive components of the flexible functions *s_j_*(X*_j_*), as in the additive model:

$$g(\mu )=\beta_{0}+s_{1}(X_{1})+s_{2}(X_{2})+\cdots +s_{p}(X_{p}).$$

This model is very flexible, but the fitting to data is much more complicated. The general local scoring algorithm is a 2-stage iterative calculation that includes the algorithms of the scoring method and backfitting.

The selection of GLIM or GAM depends on the purpose of the study. GLIM focuses on the estimation, inference, and interpretation of regression coefficients, while GAM is usually used for flexible graphical descriptions of data.

Let us focus on the distinctive features of the study on particulate matter and daily mortality. In that study, the response variable is a count variable, and the objective is inference to the coefficient for particulate matter, adjusting for temperature and humidity. Because we need the estimate of a rate ratio for the particulate matter, with flexible adjustment for other variables, a mixture of linear and smoothing parts is often used in the modeling.^22^ To partial out the long-term trend, some investigators prefer to include an arbitrary smooth function of calendar times.^23^^–^^25^ An example of such a model using the generalized additive model is:

$$\ln(\mu )=\beta_{0}+\beta_{1}\mathrm{PM}+s_{1}(\mathrm{TEMP})+s_{2}(\mathrm{HUM})+s_{3}(\mathrm{TIME}),$$

where PM is particulate matter, TEMP is temperature, HUM is humidity, and TIME is calendar time. We can estimate the risk ratio for a 10-unit increase in particulate matter as exp(10 β_1_).

## GENERALIZED ESTIMATING EQUATIONS

Most statistical methods implicitly assume that data are independent, eg, one person’s disease status is not affected by another person’s disease status. However, data may be dependent or correlated with each other, as when peak expiratory flow is measured repeatedly in the same person,^26^ when disease occurrences are observed in a family, or when a sampling unit is a cluster or a group. In environmental epidemiology, data are often semi-ecological. Exposure information is obtained from a monitoring station as a representative of a specific area, which is ecological, and disease occurrence and covariates are collected on an individual level. Disease outcomes of study participants living in a specific area tend to be correlated.

In the analysis of correlated data, the usual statistical methods yield incorrect results because they do not account for the correlation structure of the data. Generalized estimating equations (GEE)—which are an extension of GLIM—can be used to properly analyze correlated data. Consider a study of the effect of nitrogen oxides (NOx) and childhood asthma. Data from 9 participants are collected and the logistic regression model is fitted to estimate the odds ratio per unit increase of NOx, while adjusting for confounding factors. The analysis results, the test of the null exposure effect, and 95% confidence intervals for the odds ratio will be valid when the data are independent. If, instead, the data are classified into 3 areas and each area consists of 3 participants, we have to incorporate the correlation structure into the analysis. Among the possible correlation structures are an independent structure, where

$$\left(\begin{array}{ccc} 1 & 0 & 0\\ 0 & 1 & 0\\ 0 & 0 & 1 \end{array}\right),$$

an exchangeable structure, where

$$\left(\begin{array}{ccc} 1 & r & r\\ r & 1 & r\\ r & r & 1 \end{array}\right),$$

a first-order auto-regressive structure, where

$$\left(\begin{array}{ccc} 1 & r & r^{2}\\ r & 1 & r\\ r^{2} & r & 1 \end{array}\right),$$

and an unspecified structure, where

$$\left(\begin{array}{ccc} 1 & r_{1} & r_{2}\\ r_{1} & 1 & r_{3}\\ r_{2} & r_{3} & 1 \end{array}\right)$$

(*r* denotes a correlation coefficient).

When we know the true correlation structure, we can analyze the data using the weighted logistic model, where weights are a function of the true correlation structure. In epidemiologic applications, we are not entirely sure of the true correlation structure. In such cases, Liang and Zeger have proposed the use of a working correlation structure.^27^ Even when the correlation structure is misspecified, point estimates of odds ratios are unbiased, but their standard errors are biased. Hence, the test of null exposure effect and the 95% confidence intervals are not valid. To address this, they proposed using robust estimators of standard errors that are unbiased even when the correlation structure is misspecified.

GEE methods are useful for the analysis of correlated data. Since GEE is an extension of GLIM to correlated data, any type of regression model can be used to estimate the exposure effect adjusted for confounding factors. The only restriction in the application of GEE methods is that one needs appropriate statistical software to calculate the robust standard errors.

## SUMMARY

We focused on environmental (air pollution) epidemiology and reviewed some of the salient epidemiologic and statistical methods. Because case–crossover designs are variants of matched case–control studies, the use of matched analyses, such as the Mantel–Haenszel methods or conditional logistic regressions, is required. For 2-stage case–control designs, we need software with weighted logistic regressions. Most statistical software packages have programs for matched analyses and weighted regression analyses. Although GLIM, GAM, and GEE require special analyses, they are implemented in standard statistical software. With the development of statistical software, applications of the reviewed approaches are increasing dramatically. The increased use of these analytical techniques would be helpful in environmental epidemiology, and in other areas of epidemiology, as well.
